# Bioactive Calcium Silico-Phosphate Glasses Doped with Mg^2+^ and/or Zn^2+^: Biocompatibility, Bioactivity and Antibacterial Activity

**DOI:** 10.3390/antibiotics14060534

**Published:** 2025-05-22

**Authors:** Laura-Nicoleta Dragomir, Cristina-Daniela Ghiţulică, Andreia Cucuruz, Andreea Lazar, Georgeta Voicu, Sorina Dinescu

**Affiliations:** 1 Science and Engineering of Oxide Materials and Nanomaterials Department, Faculty of Chemical Engineering and Biotechnologies, National University of Science and Technology Politehnica Bucharest, 1-7 Gh. Polizu Street, Sector 1, RO-011061 Bucharest, Romania; laura.dragomir1212@upb.ro (L.-N.D.); cristina.ghitulica@upb.ro (C.-D.G.); 2 Biomaterials and Medical Devices Department, Faculty of Medical Engineering, National University of Science and Technology Politehnica Bucharest, 1-7 Gh. Polizu Street, Sector 1, RO-011061 Bucharest, Romania; andreia.cucuruz@upb.ro; 3 Biochemistry and Molecular Biology Department, Faculty of Biology, University of Bucharest, Splaiul Independenței, RO-050095 Bucharest, Romania; andreea.lazar@bio.unibuc.ro (A.L.); sorina.dinescu@bio.unibuc.ro (S.D.)

**Keywords:** bioactive glass, biomineralization, antibacterial activity

## Abstract

Bioactive glasses in the SiO_2_-CaO-P_2_O_5_ system represent emerging materials for hard-tissue-regeneration applications. This article focuses on the synthesis, characterization, and biological interaction of glasses doped with Mg^2+^ and/or Zn^2+^, with an emphasis on their effects on biomineralization, antibacterial behavior, and interactions with preosteoblasts from the MC3T3-E1 cell line. The bioglasses were synthesized using the sol-gel method, and the vitreous nature remained predominant even after thermal treatment at 600 °C for 2 h. From an in vitro perspective, the synthesized bioglasses demonstrated strong cell adhesion and proliferation (notably in the case of Mg^2+^ doping), low cytotoxicity, and antibacterial properties (especially in Zn^2+^-doped samples). Additionally, the simultaneous doping with Mg^2+^ and Zn^2+^ of the bioactive glass matrix is a prospective strategy for developing biomaterials with a “dual” biological characteristics–both osteoinductive and antibacterial.

## 1. Introduction

In recent decades, vitreous bioactive materials (bioglasses or biovitroceramics) have undergone significant development, being recognized for their ability to interact with biological tissues and to induce bone regeneration through the formation of a superficial hydroxyapatite (HA) layer, similar to the mineral matrix of natural bone or as multifunctional drug delivery systems [1,2,3,4,5,6,7,8,9]. Bioactive glasses, particularly those in the SiO_2_–CaO–P_2_O_5_ system, offer a versatile platform for the development of advanced biomaterials due to their composition, which closely resembles that of bone tissue, and the possibility of modifying their chemical structure by doping with therapeutic ions (e.g., Ag^+^, Cu^2+^, Mn^2+^, Ce^4+^, Mg^2+^, Zn^2+^, Sr^2+^), which can favorably influence the biological behavior of the glass, such as preventing the growth and reproduction of bacteria [7,10,11,12].

Magnesium is the fourth most common intracellular cation in the body and plays an essential role in bone homeostasis, being involved in enzymatic activation, cell proliferation, and the mineralization of the extracellular matrix [10,13,14,15,16,17,18,19]. It has been shown that Mg^2+^ can positively influence the topology of the glass network, facilitating the controlled dissolution and gradual release of bioactive ions into the physiological environment, which contributes to the creation of a microenvironment favorable for bone regeneration [16,17].

Zinc, in turn, is an essential trace element, known for its antibacterial properties and its role in stimulating osteogenesis and inhibiting osteoclastogenesis [20,21,22,23,24,25,26,27]. Studies have shown that Zn^2+^ can induce bacteriostatic effects against common strains from both the Gram-negative and Gram-positive spectrum involved in nosocomial infections, such as *Staphylococcus aureus* and *Escherichia coli*, making it a valuable dopant in orthopedic and dental applications [22,24,27,28,29]. Additionally, there is a hypothesis that Zn^2+^ disrupts intracellular metal homeostasis in bacteria, leading to increased oxidative stress generated by reactive oxygen species (ROS) [30,31].

The simultaneous integration of Mg^2+^ and Zn^2+^ into a bioactive glass matrix is a promising strategy for developing biomaterials with complex biological behavior. The synergistic release of these ions may lead to faster bone regeneration while concurrently reducing the risk of bacterial colonization on the implant surface [12].

Based on these considerations, the present study aimed to investigate the effects of Mg^2+^ and/or Zn^2+^ doping on the structural, bioactive, and antibacterial properties of glasses based on the CaO–SiO_2_–P_2_O_5_ system. In addition, another aim was to add a higher concentration of the dopant, to increase biological activity, but without significantly affecting the vitreous structure via the crystallization process.

## 2. Results

### 2.1. Synthesis of Bioglass Powders

**Figure 1:**
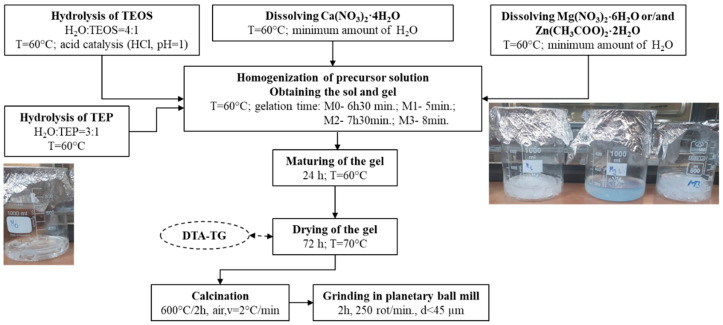
Synthesis scheme of bioglass powders.

The weight compositions of the studied glasses, from the CaO-SiO_2_-P_2_O_5_-(ZnO, MgO) system, are presented in Table 1, and they were synthesized using the sol-gel method (Figure 1). The gelation times ranged from 5 min to 7 h and 30 min, with the observation that the addition of ZnO led to a decrease in the gelation time, whereas MgO had the opposite effect. The appearance of the gels after 24 h at 60 °C is shown in Figure 1.

### 2.2. Characterization of the Dried Gels

**Figure 2:**
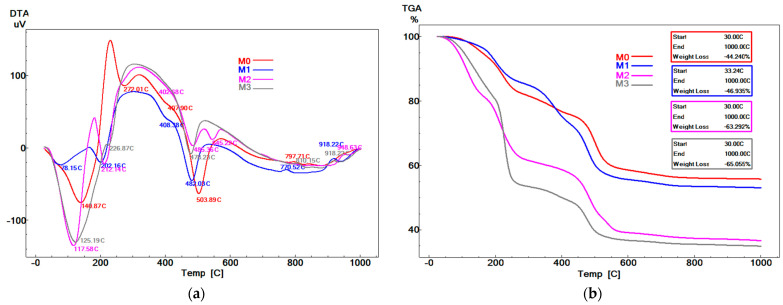
Complex thermal analysis of gels aged and dried for 72 h at 70 °C: (**a**)—DTA curves; (**b**)—TG curves.

The dried gels were characterized via thermal analysis (DTA-TG) and X-ray diffraction (XRD). From Figure 2, it can be observed that the DTA curves show between four and five endothermic effects up to 600 °C, accompanied by mass loss on the TG curves.

These events can be associated with various decomposition processes, such as evaporation of the alcoholic fraction resulting from the synthesis (between 78–141 °C), decomposition of hydroxylated or phosphate phases (between 202–272 °C), decomposition of hydrated hydroxylated or phosphate phases (also between 202–272 °C), and decomposition of calcium nitrate hydrate (with *x* H_2_O molecules, where *x* < 4) in the range of 402–545 °C.

Above 600 °C, the TG curves show no significant mass changes, while the DTA curves display two exothermic effects with peaks in the ranges of 770–810 °C and 918–948 °C, which can be attributed to the crystallization of mineralogical phases characteristic of the studied oxide system. Therefore, to eliminate all decomposition processes, the dried gels were heat-treated at 600 °C for 2 h (heating rate: 2 °C/min), and the resulting materials were ground in a planetary ball mill for two hours, after which the residue on the 45 μm sieve was 0%.

**Figure 3:**
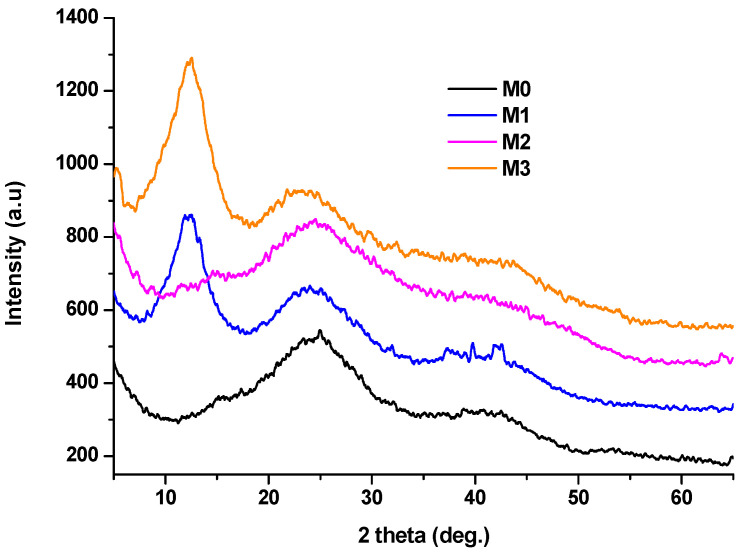
XRD patterns of gels aged and dried for 72 h at 70 °C.

These findings are supported by the XRD patterns in Figure 3, where the presence of broad diffraction halos indicates a very low degree of crystallinity.

These halos correspond to the diffraction of reprecipitated calcium nitrate hydrate with *x* water molecules (*x* < 4), according to the standard JCPDS files 070-0204 and 019-0255. Additionally, the presence of Zn^2+^ appears to induce a higher tendency for reprecipitation (two hallows in cascade), where the gels containing Zn^2+^ (M1 and M3) appear opalescent.

### 2.3. Characterization of the Calcined Powders

**Figure 4:**
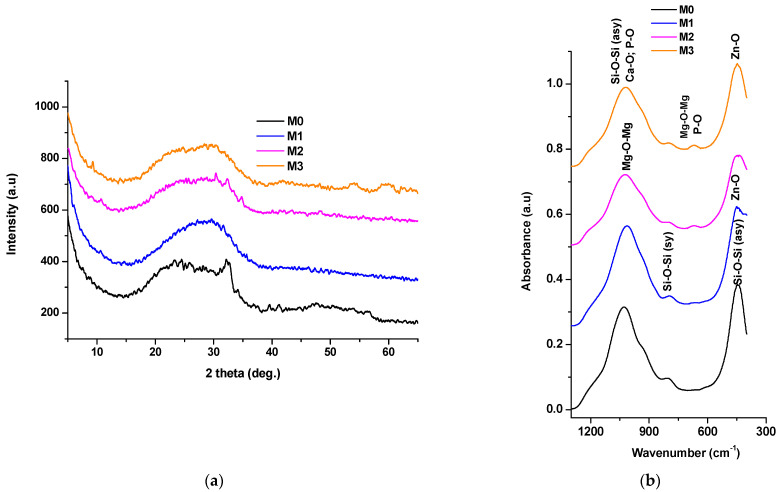
XRD patterns (**a**) and FTIR spectra (**b**) of the materials obtained via heat treatment at 600 °C for 2 h of the gels dried for 72 h at 70 °C.

**Figure 5:**
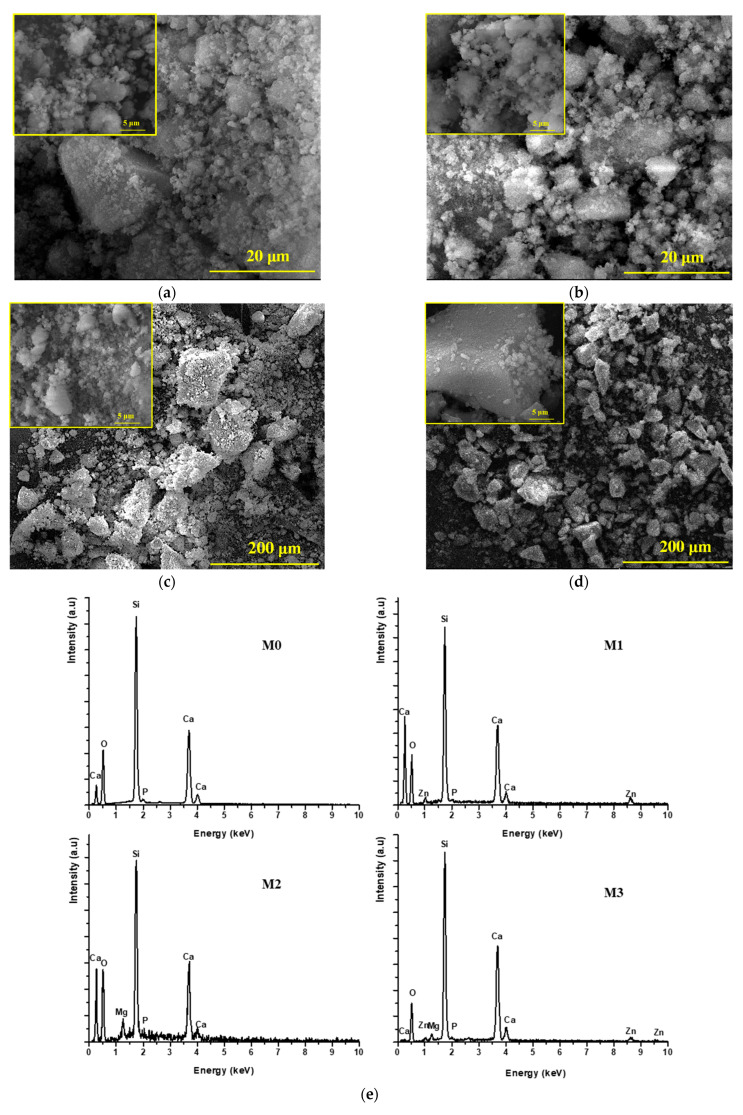
SEM images ((**a**)–M0; (**b**)–M1; (**c**)–M2; (**d**)–M3) and EDAX spectra (**e**) of the synthesized vitreous powders.

The characterization of the powders obtained by calcining the dried gels at 600 °C for 2 h was performed using X-ray diffraction (XRD), Figure 4a, Fourier-transform infrared spectroscopy (FTIR), Figure 4b, and scanning electron microscopy (SEM) accompanied by energy-dispersive X-ray spectroscopy (EDAX), Figure 5.

From Figure 4a, it can be observed that even after heat treatment, the vitreous nature of the materials is preserved, as evidenced by the presence of a halo in the low-angle range (below 35°); in the case of sample M0, diffraction interferences characteristic of hydroxyapatite are visible within the halo, according to standard JCPDS file 084-1998. These findings are supported by the FTIR results presented in Figure 4b, where absorption bands characteristic of the [SiO_4_]^4−^ group (447–449 cm^−1^; 795–800 cm^−1^; 1014–1027 cm^−1^) and bonds such as Ca–O–Si (920 cm^−1^), Zn–O (437–439 cm^−1^), Mg–O–Mg (668–673 cm^−1^, 1018 cm^−1^), and P–O (620 cm^−1^, 939–947 cm^−1^) can be observed, depending on the oxide composition of the samples.

Morphologically, the SEM images in Figure 5 show that the analyzed powders are composed of polyhedral particles with sharp edges and corners, a morphology typical of vitreous powders. Additionally, it can be seen that these particles are aggregates made up of mesometric particles (larger than 100 nm), and the EDAX spectra confirms the elemental composition of the studied materials.

### 2.4. In Vitro Characterization of the Synthesized Powders

**Figure 6:**
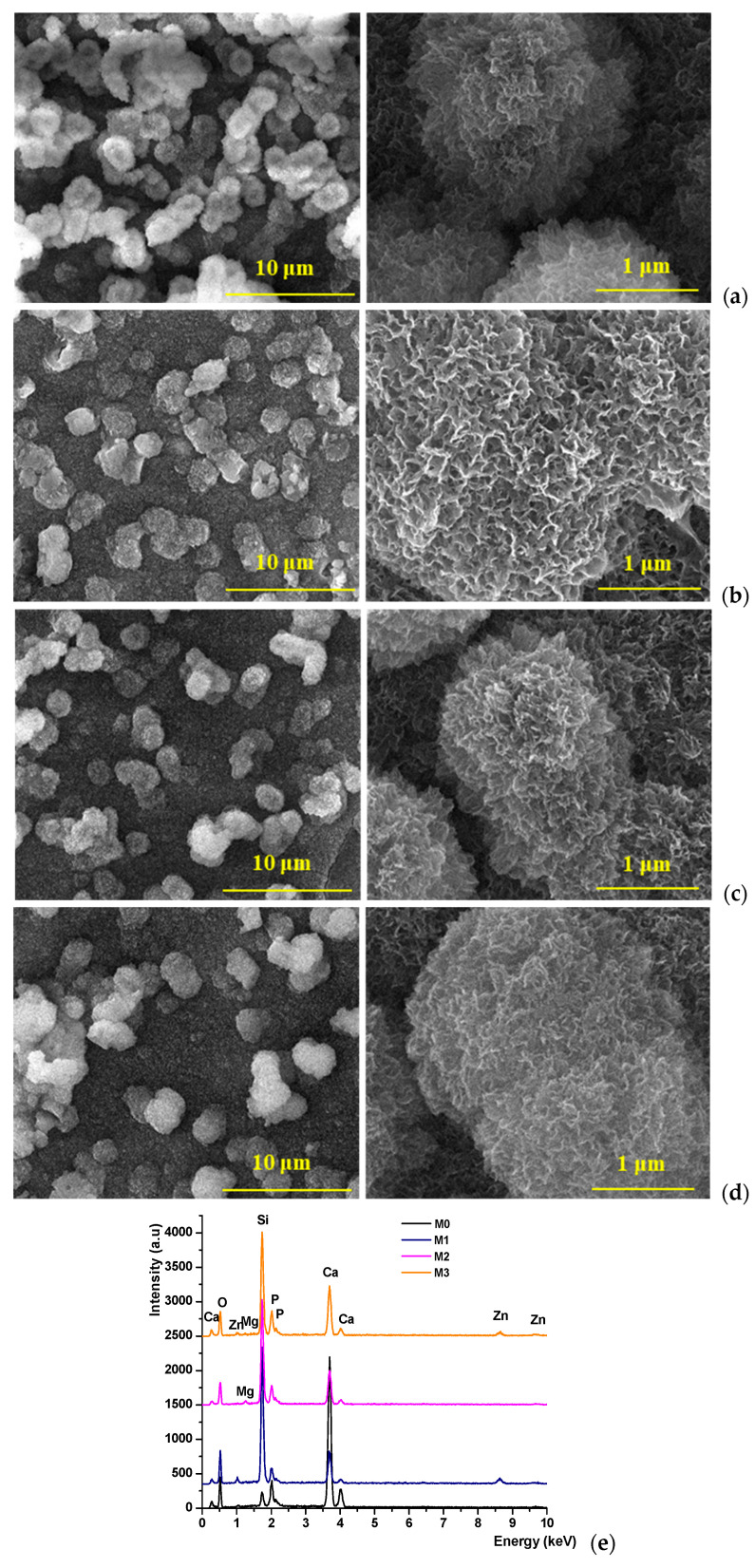
SEM images ((**a**)–M0; (**b**)–M1; (**c**)–M2; (**d**)–M3) and EDX spectra (**e**) for the synthesized glass powders immersed in SBF for 14 days, at 37 °C.

All glass powders were tested for bioactivity by immersion in simulated body fluid (SBF) for 14 days at 37 °C. From the obtained scanning electron microscopy (SEM) images, Figure 6, it can be observed that all samples underwent mineralization, through the formation of a layer composed of spherical-shaped formations made up of plate-like structures, indicating good interaction with the SBF.

According to literature data, this morphology is characteristic of apatite phases, and the EDAX spectra confirm their formation [32,33]. Moreover, it can be noted that the degree of mineralization is not influenced by the ZnO and MgO content in the composition.

**Figure 7:**
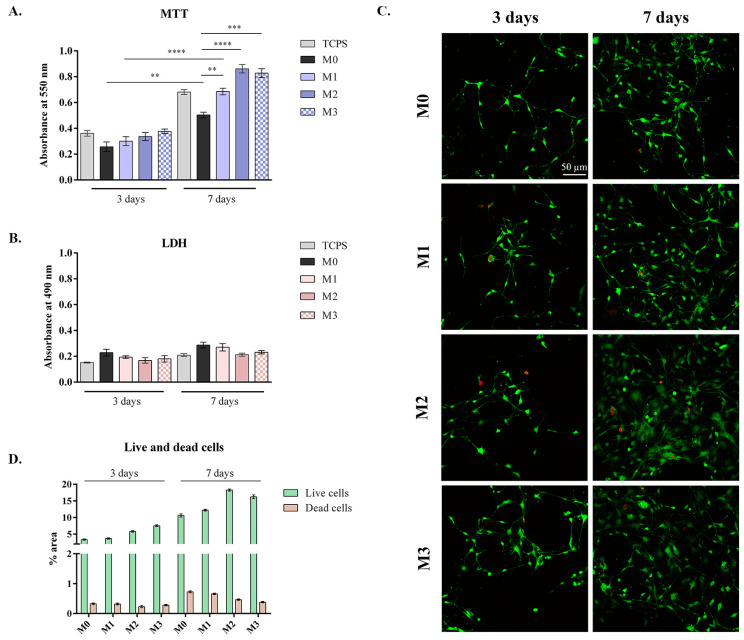
(**A**). The viability and proliferation profile of MC3T3-E1 preosteoblasts in contact with the bioactive glasses (*p* < 0.01 **; *p* < 0.001 ***; *p* < 0.0001 ****), compared to a standard TCPS control. (**B**) Cytotoxicity of the materials assessed by means of the LDH assay. Similar levels of cytotoxicity on all tested composites were observed, with statistically insignificant differences, suggesting good biocompatibility. (**C**) Qualitative evaluation of the viability and proliferation of preosteoblasts in contact with the bioactive glasses by means of the Live/Dead assay. Live cells are labeled with calcein and stained green, while dead cell nuclei are stained red with EtBr. Scale bar for all images, 50 µm. (**D**) Quantification of green fluorescence (live cells) levels and red fluorescence (dead cell nuclei) levels in all composites.

Cell viability and proliferation were quantitatively evaluated during one week of in vitro cell culture in the presence of the bioactive calcium silicophosphate glasses doped with Mg^2+^ and/or Zn^2+^ using an MTT assay, which measures cell metabolic activity (Figure 7A).

All tested composites displayed good cell viability after 3 days of culture, without statistically significant differences between them. However, after one week of culture, the viability of MC3T3-E1 preosteoblasts was significantly higher on the glasses enriched with minerals, compared to the control material and the standard TCPS control. In this regard, significant differences were observed between the bioactive glasses supplemented with Zn^2+^ (M1, *p* < 0.01), Mg^2+^ (M2, *p* < 0.0001), or both (M3, *p* < 0.001) and the M0 control, as well as TCPS, with the addition of Mg^2+^ and/or Zn^2+^ favoring cell viability.

Moreover, higher cell proliferation was observed from 3 to 7 days of culture for all tested composites, but the most significant difference was registered for the bioactive glasses doped with minerals (*p* < 0.0001), suggesting that the addition of Mg^2+^ and/or Zn^2+^ has a positive effect upon cell growth and development. Additionally, better results were obtained for the glasses enriched with Mg^2+^ (M2 and M3), when compared to the Zn^2+^ doped material (M1, *p* < 0.05), which indicates a more potent role for Mg^2+^ in supporting optimal cell growth. In conclusion, these results indicate that the bioactive glasses doped with minerals are biocompatible with preosteoblasts, with the addition of Mg^2+^ and/or Zn^2+^ having a positive effect on cell viability and proliferation.

The potential cytotoxicity of the calcium silicophosphate glasses, enriched or not with Mg^2+^ and/or Zn^2+^, was tested by measuring the LDH levels in the culture media during one week of in vitro cell culture, as shown in Figure 7B. After 3 days, similar LDH levels were registered on all tested composites, without significant differences between them. After 7 days of culture, these levels increased slightly, however not significantly, compared to the ones measured after the first time point. Therefore, the addition of minerals did not induce significant cytotoxic effects on MC3T3-E1 preosteoblasts in our experimental conditions.

After 3 and 7 days of culture, Live/Dead staining was performed on all tested composites, followed by confocal microscopy analysis, which allowed for the evaluation of cell viability, morphology, and proliferation. The ratio between live and dead cells was strongly positive in all samples, at both time points, similar to the quantitative results obtained from MTT and LDH assays, suggesting no significant negative impact on cell behavior (Figure 7C). As such, after 3 days of culture, a similar number of live cells can be observed on the composites, with no distinguishable difference between them. However, after 7 days of culture the bioactive glasses doped with minerals, particularly Mg^2+^ (M2), supported higher cell viability rates than simple control glass (M0) (~2 times higher), whereas the Zn^2+^-doped bioglass (M1) showed a similar profile compared to the control M0. The cumulative effect of the Mg^2+^ and Zn^2+^ doped in the same glass (M3) exhibited an average cell response in terms of cell viability when compared to M0. These results suggest that cellular growth is encouraged by the addition of Zn^2+^ and/or Mg^2+^.

In addition, higher proliferation from 3 to 7 days was registered on the materials enriched with minerals compared to M0 (~3 times), suggesting a good environment for cell viability and proliferation.

Overall, the quantification of green fluorescence (live cells) levels and red fluorescence (dead cell nuclei) levels in all composites corroborated the qualitative results obtained through microscopy images, with a much higher amount of green fluorescence (5–20× higher) than red fluorescence (Figure 7D).

Therefore, the biocompatibility evaluation revealed low cytotoxicity, good viability, and proliferation for all tested composites seeded with MC3T3-E1 preosteoblasts; however, better results were obtained for the bioactive glasses doped with natural elements, Zn^2+^ and/or Mg^2+^, which showcased higher cell viability and enhanced proliferation, as well as better distribution on the surface. These observations are in accordance with other similar studies, where it has been shown that the presence of Zn^2+^ and/or Mg^2+^ in the materials is beneficial because these elements play important roles in bone cell regeneration, especially osteoblast proliferation, facilitating cytocompatibility, cell adhesion and mineralization both in vitro and in vivo [34,35,36,37,38]. Furthermore, by incorporating Zn^2+^, bioactive glasses can prevent bacterial proliferation, exhibit anti-inflammatory properties, and influence bioactivity, enhancing integration with surrounding tissues when incorporated into scaffolds for bone tissue engineering applications [39,40].

**Figure 8:**
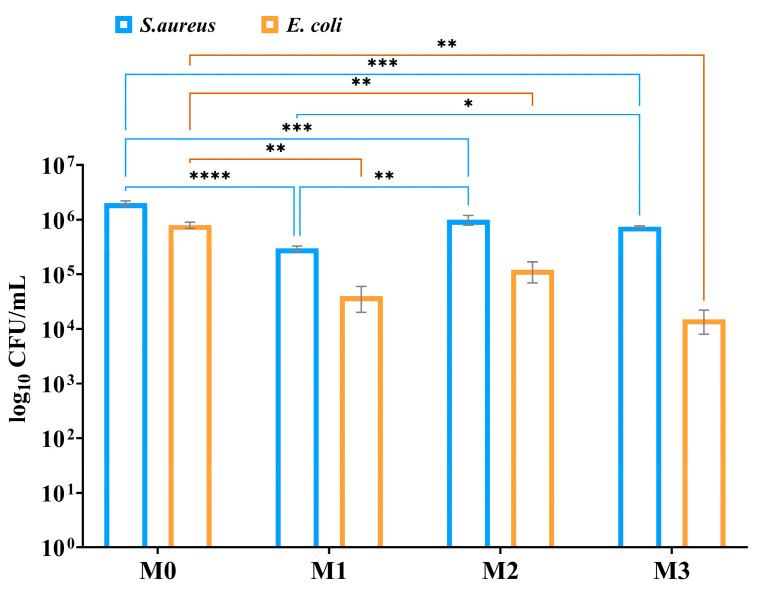
The antibiofilm effect of bioactive glasses after 24 h of incubation in liquid media for tested strains was compared using two-way ANOVA and Tukey’s multiple comparisons tests. The results were considered statistically significant (* *p* < 0.026; ** *p* < 0.003; *** *p* ≤ 0.0004; **** *p* < 0.0001).

It can also be observed that the vitreous materials, especially those containing Zn^2+^ (M1 and M3), exhibit antibacterial activity, in agreement with literature data [41,42], Figure 8, preventing the formation of a bacterial biofilm.

## 3. Materials and Methods

For synthesis from Figure 1, chemically pure reagents were used: tetraethyl orthosilicate (TEOS, ≥99%, Sigma-Aldrich, Darmstadt, Germany), calcium nitrate tetrahydrate (Ca(NO_3_)_2_·4H_2_O, ≥99%, Sigma-Aldrich, Darmstadt, Germany), triethyl phosphate ((C_2_H_5_O)_3_PO, TEP; >98%, Sigma-Aldrich, Darmstadt, Germany), magnesium nitrate hexahydrate (Mg(NO_3_)_2_·6H_2_O, 99%, Sigma-Aldrich, Darmstadt, Germany) and zinc acetate dihydrate (Zn(CH_3_COO)_2_·2H_2_O, ≥98%, Sigma-Aldrich, Darmstadt, Germany).

The chemical–mineralogical composition and crystallinity of the dried gels and the resulting powders were evaluated via X-ray diffraction (XRD), using a Shimadzu XRD 6000 diffractometer (Shimadzu, Kyoto, Japan), with Ni-filtered Cu Kα radiation (λ = 1.5406 Å), in a 2θ range of 5 to 65°, with a scanning speed of 2°/min and a step size of 0.02 min/step. Additionally, to determine the calcination temperature for the dried gels, complex thermal analysis was performed using a Shimadzu DTG-60 analyzer (Shimadzu, Kyoto, Japan); the analyses were carried out in the temperature range of 30–1000 °C, with a heating rate of 10 °C/min, in air.

The structural characteristics of the glass powders were analyzed via Fourier-transform infrared (FT-IR) spectroscopy using a Thermo iN10-MX instrument (Thermo Fisher Scientific, Waltham, MA, USA). FT-IR spectra were obtained in absorbance mode over the 1300–300 cm^−1^ range, with a spectral resolution of 4 cm^−1^.

The morpho-structural characterization of the precursor powders and ceramic materials was performed via scanning electron microscopy (SEM) using a Quanta Inspect F50 FEG scanning electron microscope, with a resolution of 1.2 nm (Thermo Fisher, Eindhoven, The Netherlands). To enhance conductivity under the electron beam, the samples were coated with a thin layer of gold.

Taking a biological approach, the synthesized vitreous materials were tested in vitro for biocompatibility and bioactivity. For these analyses, the vitreous powders were uniaxially pressed into 13 mm-diameter pellets at approximately 2 tons of force, while the simulated body fluid (SBF, pH = 7.4) was freshly prepared as described by Kokubo et al. [43].

The bioactivity of the glass powders was evaluated by immersing the samples in simulated body fluid (SBF, pH = 7.4) for 14 days at 37 °C, without refreshing it up to term. Triplicate samples were soaked in SBF at a concentration of 1 mg/mL. Prior to drying the pellets for 12 h at 60 °C, they were gently rinsed with distilled water. Finally, the composition and morphology of the immersed samples were analyzed using SEM/EDX.

In order to evaluate the biocompatibility of bioactive calcium silico-phosphate glasses doped with Zn^2+^ (M1), Mg^2+^ (M2), or both (M3), viability and cytotoxicity assays were performed after 3 and 7 days of in vitro cell culture in standard conditions (37 °C, 5% CO_2_, humidity), in comparison to the control material (M0) without these minerals. Cell viability and proliferation were determined using a qualitative Live/Dead fluorescent staining and quantitative MTT assay, while the glass cytotoxicity was determined using an LDH assay.

*Cell-scaffold culture model*: The biocompatibility evaluation was performed in vitro against preosteoblasts from the MC3T3-E1 cell line, which were cultured in DMEM media supplemented with 10% FBS and 1% antibiotic antimycotic solution. First, the glasses were sterilized for 6 h on each side and washed several times in a phosphate-buffered saline solution supplemented with 4% antibiotics. Afterwards, cells were seeded on their surface at a density of 2.5 × 10^4^ cells/cm^2^ and kept under standard culture conditions for a week (5% CO_2_, 37 °C, in a humidified atmosphere). The culture plates were regularly observed with a phase contrast microscope, and the biocompatibility assays were performed at 3 and 7 days post-seeding.

*MTT assay*: Cell viability and proliferation were evaluated using methylthiazolyldiphenyl tetrazolium bromide (MTT, Sigma/Merck, Steinheim, Germany). An MTT solution of 1 mg/mL was prepared in cell culture media without fetal bovine serum, and after 4 h of incubation in standard conditions, isopropanol was used to dissolve the formazan crystals formed by the metabolic active cells found on the materials. The absorbance of the violet solution was measured at 550 nm using the FlexStation3 spectrophotometer (Molecular Devices, San Jose, CA, USA). The values obtained were directly proportional to the number of live cells.

*LDH assay*: The cytotoxic potential of the enriched bioactive glasses was determined using the TOX7 kit (“In Vitro Toxicology Assay kit, Lactic Dehydrogenase Based”, Sigma/Merck, Steinheim, Germany), which quantifies the levels of the LDH enzyme released by cells without an intact cell membrane. The test was performed according to the instructions provided by the manufacturer (media were collected from the culture wells, mixed with the components of the kit, and then incubated for 15 min in the dark at room temperature) and the optical density of the final solution was measured at 490 nm using a FlexStation3 (Molecular Devices, San Jose, CA, USA). The values obtained were directly proportional to the number of dead cells.

*Live/Dead assay*: To qualitatively evaluate cell viability, the Live/Dead Kit (Invitrogen, Life Technologies, Foster City, CA, USA) was employed, staining live cells (green) with calcein acetoxymethyl (AM) and the nuclei of non-viable cells (red) with ethidium homodimer-1 (EthD-1). Following the manufacturer’s protocol, the staining solution was prepared and applied to the samples, which were then incubated in the dark for one hour at room temperature. Cell imaging was performed using a laser-scanning confocal microscope (Nikon A1/A1R Confocal Laser Microscope System, Nikon Instruments Inc., Melville, NY, USA), and images were acquired using NIS-Elements software (Nikon Instruments Inc., version 5.11 64-bit, Melville, NY, USA). The quantification of fluorescence intensity for live and dead cells was performed with ImageJ software (version 1.x bundled with 64-bit Java 8).

*Statistical analysis*: All tests were performed in triplicate, and data are expressed as the mean ± SD, using GraphPad Prism Software 9.0 (Graph Pad Software Inc., San Diego, CA, USA). The statistical validity of the results was assessed using the one-way ANOVA method and Bonferroni post-test, with significant statistical differences being considered for *p* < 0.05. For fluorescence quantification, data are expressed as the mean ± SD for n = 10 fields of view/sample.

The antibacterial evaluation of the vitreous powder pellets was assessed in vitro against two bacteria, *S. aureus* (Gram-positive) and *E. coli* (Gram-negative), which were utilized in this study The strains are maintained as glycerol stocks at −80 °C in the culture collection. Glycerol stocks were streaked on nutritive agar for bacterial strains to obtain 24 h cultures to be used for all further studies.

The formation of microbial biofilms was assessed using a static biofilm model over a 24 h period, following a protocol reported in the literature [44,45,46]. Vitreous powder pellets were aseptically placed into sterile 24-well plates containing 1 mL of liquid medium, which was then inoculated with 10 µL of a 0.5 McFarland suspension, as described in the planktonic growth method. The plates were incubated at 37 °C for 24 h. Afterwards, each pellet was gently rinsed with 1 mL of PBS to eliminate non-adherent microbial cells and then transferred to 1 mL of sterile PBS in an Eppendorf tube. The samples were vortexed vigorously for 20 s and sonicated for 10 s to detach bacteria embedded within the biofilm on the pellet surfaces. The resulting microbial suspension was serially diluted ten-fold, and aliquots from each dilution were plated in triplicate on nutrient agar. The inoculated plates were incubated at 37 °C for 24 h to allow colony formation, which was then used to determine CFU/mL values, representing the viable biofilm-associated cells recovered from the vitreous powder surfaces.

All experiments were performed in triplicate, including three independent biological replicates and three technical replicates for each condition tested. Results are expressed as the mean ± standard deviation (SD), where appropriate. Standard deviations have been added to the graphs wherever relevant. Statistical significance was evaluated using one-way or two-way ANOVA, followed by either Tukey’s or Dunnett’s post hoc tests, considering *p*-values below 0.05 as statistically significant. All statistical analyses were conducted using GraphPad Prism version 10.4.1 (GraphPad Software, San Diego, CA, USA). These methods enhance the reproducibility, transparency, and reliability of the reported findings.

## 4. Conclusions

The results of this study demonstrate that the synthesized calcium silico-phosphate bioactive glasses, especially those doped with magnesium (Mg^2+^) and/or zinc (Zn^2+^), possess both excellent biocompatibility and bioactivity. The Mg^2+^-doped samples (particularly M2 and M3) significantly enhanced cell viability and the proliferation of MC3T3-E1 preosteoblasts, indicating their suitability for bone tissue engineering applications. Zn^2+^-containing glasses (M1 and M3) showed clear antibacterial effects, successfully inhibiting biofilm formation by pathogenic bacteria.

The dual doping strategy (Mg^2+^ and Zn^2+^ together) offered a promising balance between osteoinductive properties and antibacterial performance, supporting the development of multifunctional biomaterials for infection-resistant bone implants. Therefore, the incorporation of these therapeutic ions into the glass matrix provides a valuable approach for improving the biological functionality of bioactive glasses, with significant potential in regenerative medicine and implantology.

Building on the promising biological and antibacterial properties observed, future research on these Mg^2+^ and Zn^2+^-doped calcium silico-phosphate bioactive glasses could focus on their integration into 3D-printed scaffolds or coatings for metallic implants to enhance mechanical stability and osteointegration.

## Figures and Tables

**Table 1:** Oxide composition of the studied glass (wt.%).

Sample	SiO_2_	CaO	P_2_O_5_	ZnO	MgO
M1	65	26	4	5	0
M2	65	26	4	0	5
M3	65	26	4	2.5	2.5
M0	68.42	27.36	4.22	0	0

## Data Availability

The data presented in this study are available from the corresponding author upon reasonable request.
